# The Editor's Letter-Box

**Published:** 1915-11-13

**Authors:** 


An Average Practitioner.

Harley Street, W., November 9, 1915.

Sir,—Many thanks for letting me see an advance proof of your correspondent's letter. I am sorry he does not like my article. Had the contribution been from his pen it would doubtless have been a different, and probably a better one. I shall try to profit by his instruction. Unfortunately, some aspects of the subject which appeal to me do not interest him, and he condemns my article because it is not a treatise.

Quite unintentionally, I am sure, he misrepresents me when he suggests that I believe the profession to be ignorant of the risk of heart disease in the rheumatism of early life. On the contrary, my argument is that with this knowledge there is the opportunity of warning parents in advance of the risks which their children run, and the hope that in this way the frequency of cardiac disease may be lessened. If "An Average Practitioner" says that this practice is usually adopted, I am glad to hear it, but I have much evidence to the contrary.

I will not attempt to dispute with your correspondent about causation, for though we both allow our ignorance, he has more confidence than I can claim. He is ready to say that cancer and sunspots do not arise from one and the same cause, while he admits that he does not know the cause either of one or the other. Personally, I should hesitate at the proposition; it reminds me of the schoolboy who affirmed that "things which are impossible are equal to one another."—I am, etc.,
THE WRITER OF THE ARTICLE.

